# Supplementary figures and images for: One N-glycan regulates natural killer cell antibody-dependent cell-mediated cytotoxicity and modulates Fc γ receptor IIIa/CD16a structure
Source: eLife. 2024 Oct 25;13:RP100083. doi: 10.7554/eLife.100083 (PMC11509673; doi:10.7554/eLife.100083)

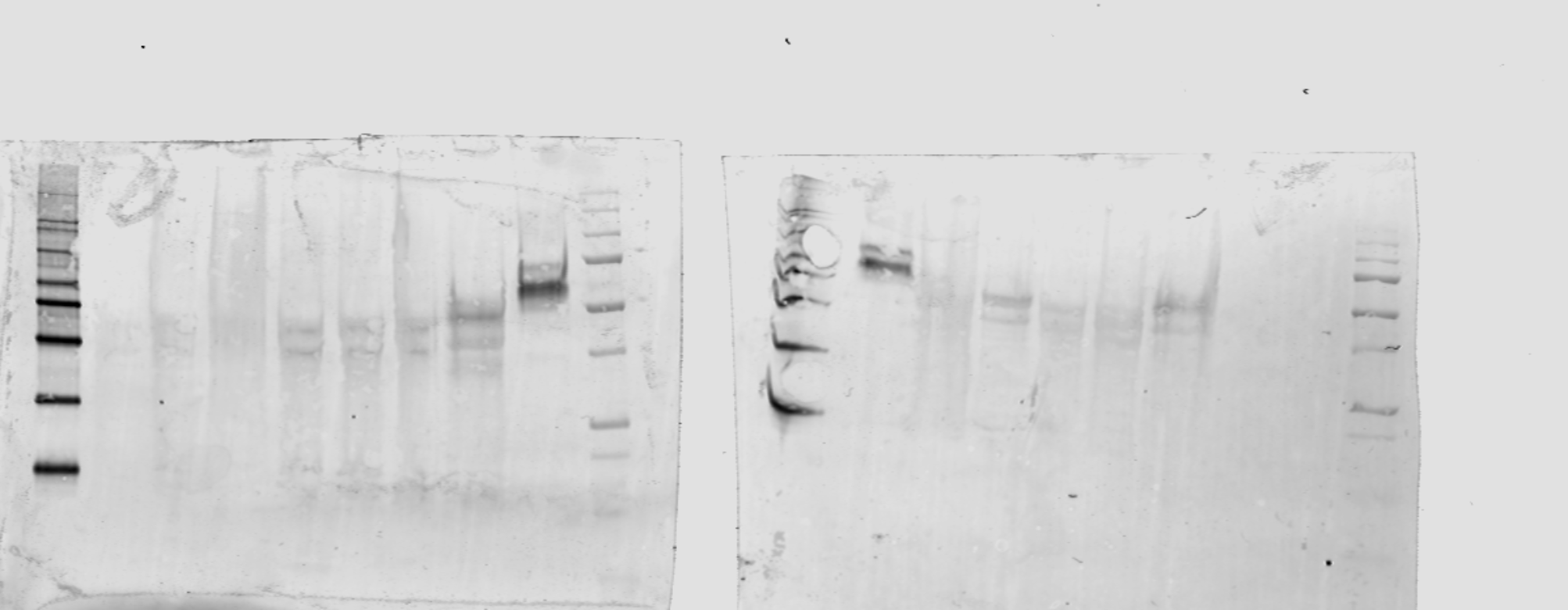

Supplement: Figure 1—figure supplement 1—source data 1. [file elife-100083-fig1-figsupp1-data1.zip › Figure S1 source data 1/Figure S1 AB source data .png]

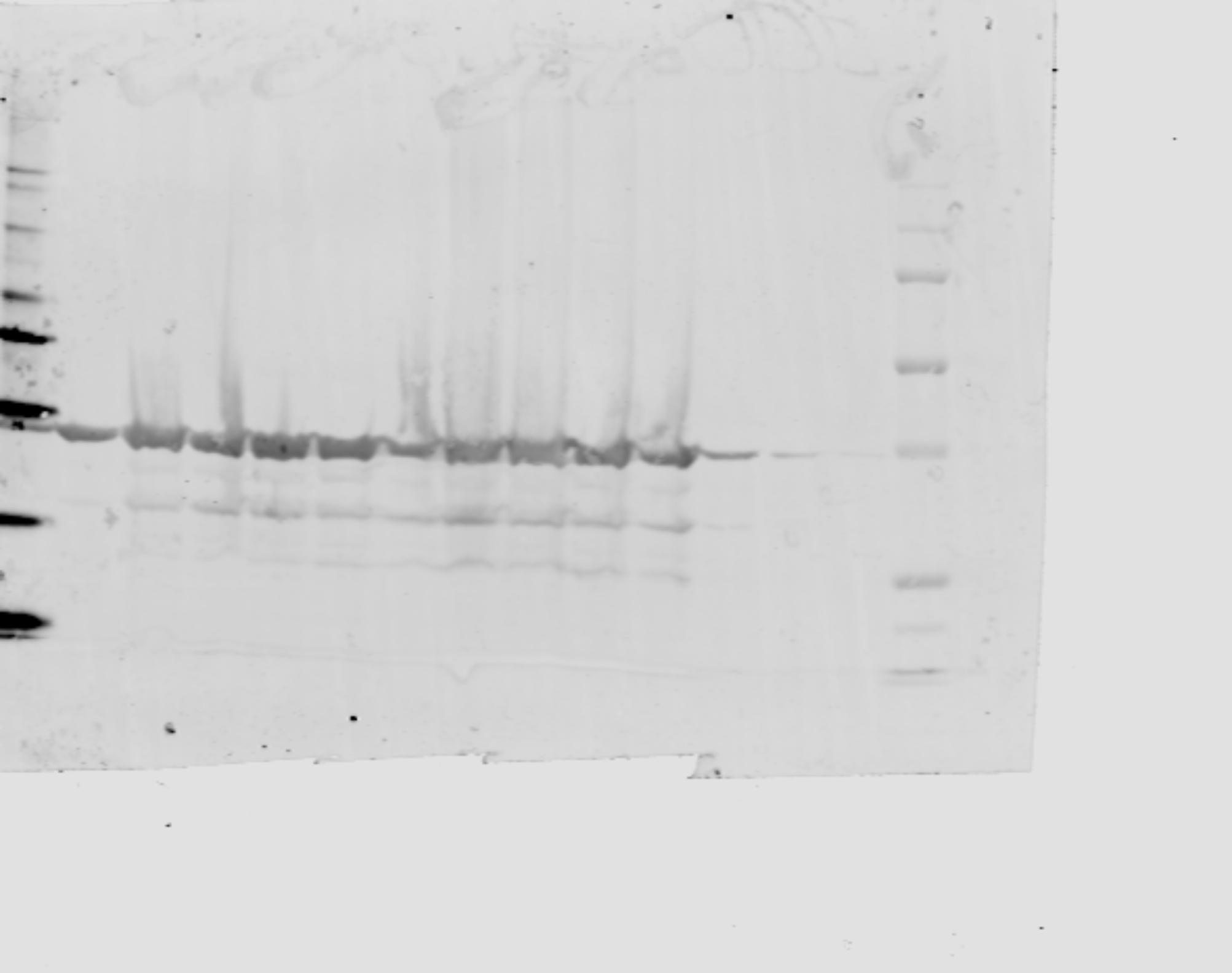

Supplement: Figure 1—figure supplement 1—source data 1. [file elife-100083-fig1-figsupp1-data1.zip › Figure S1 source data 1/Figure S1 C source data.png]

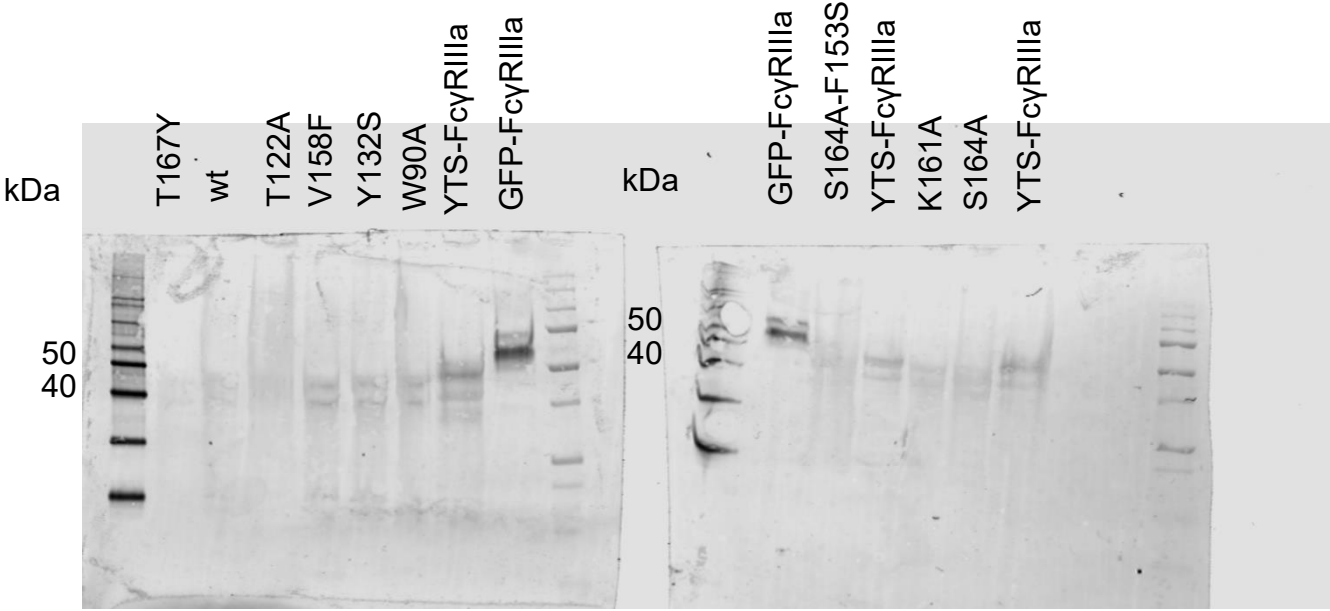

kDa

YTS-FcγRIIIa  
wt  
W90A  
Y132S  
T122A  
V158F  
T167Y  
S164A  
K161A  
S164A-F153S

40

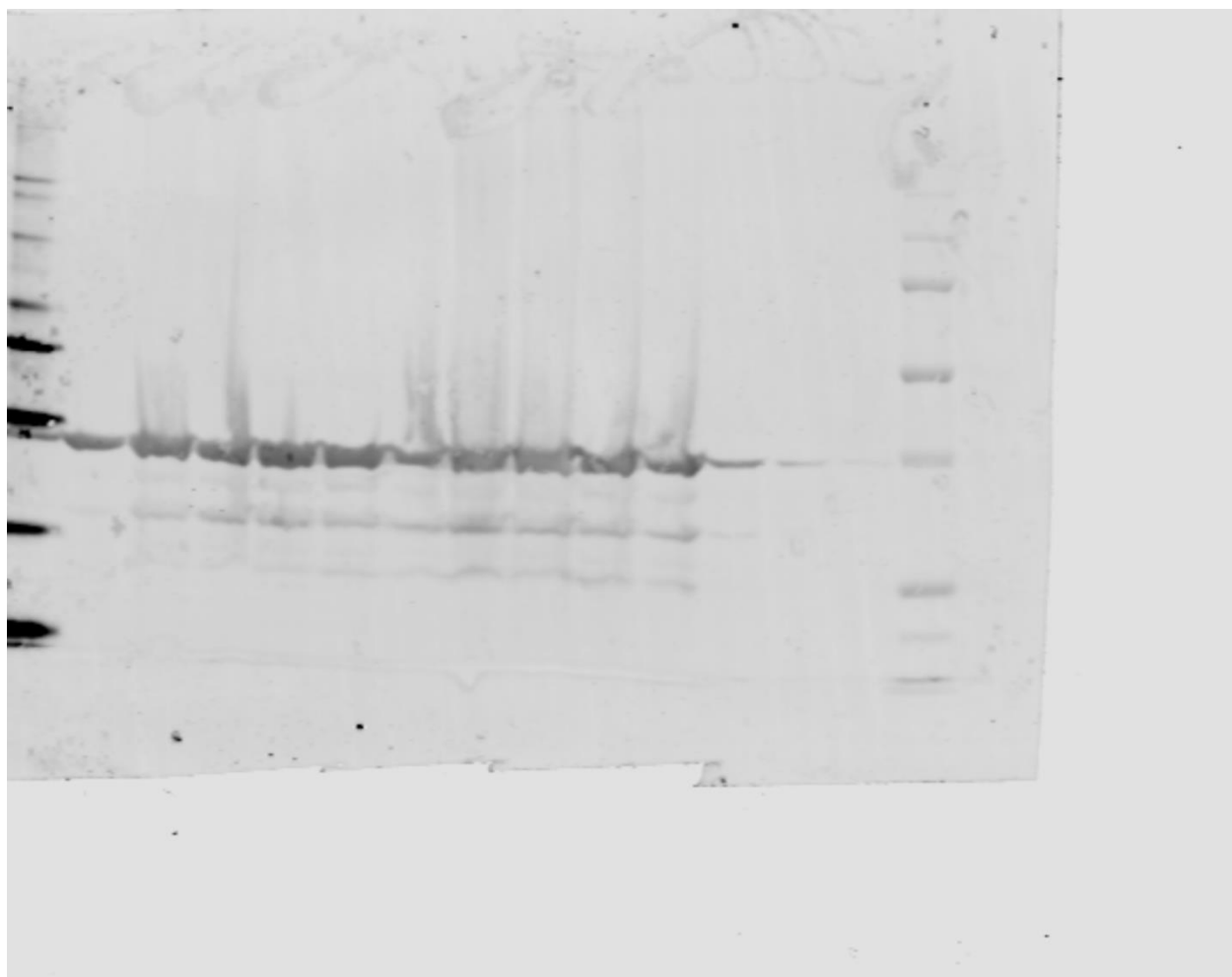

Supplement: Figure 1—figure supplement 1—source data 2. [file elife-100083-fig1-figsupp1-data2.zip › Figure S1 source data 2/Figure S1 source data 2.pdf]

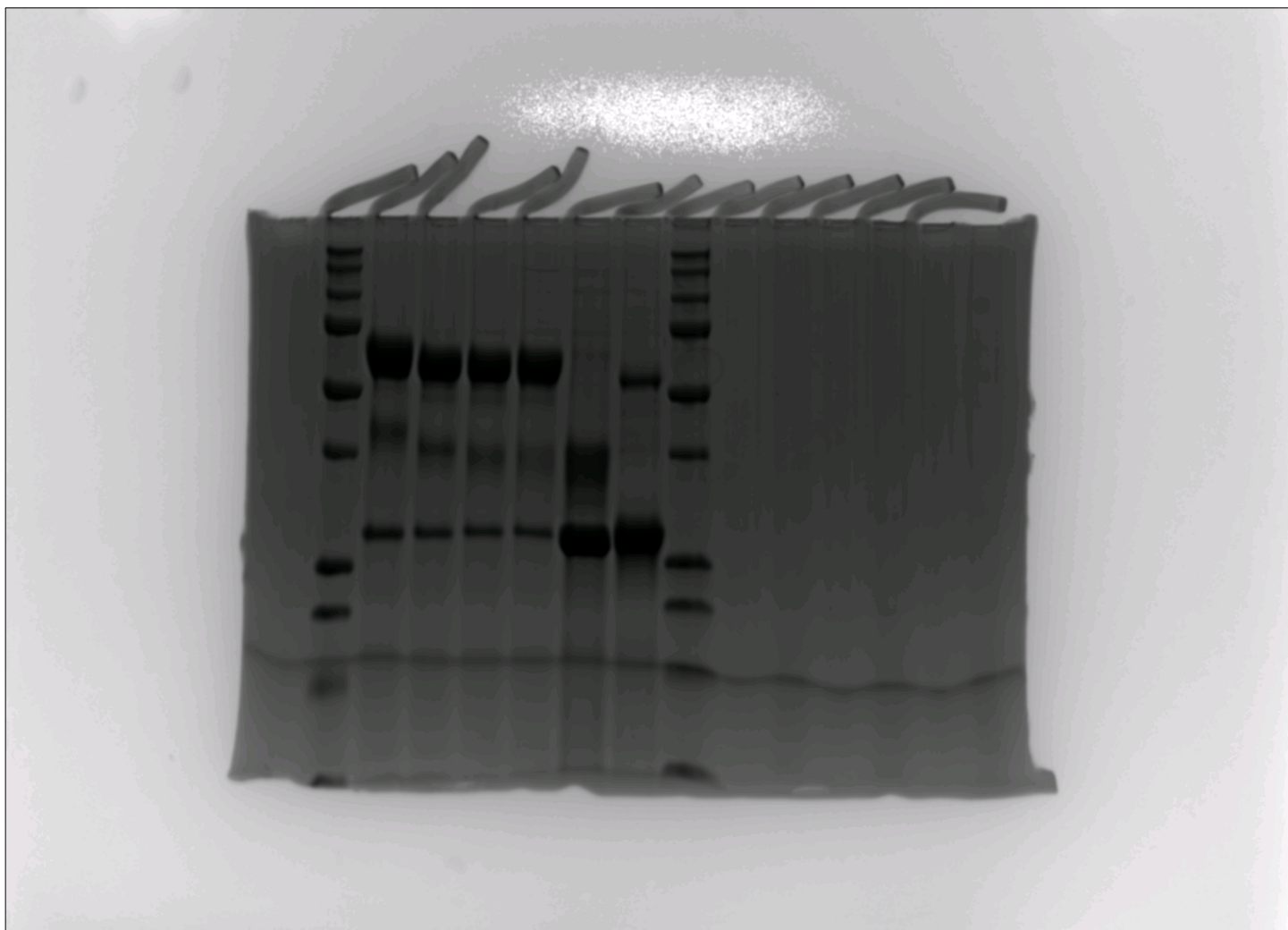

Supplement: Figure 2—figure supplement 1—source data 1. [file elife-100083-fig2-figsupp1-data1.zip › Figure S2 source data 1/Figure S2 source data 1.pdf]

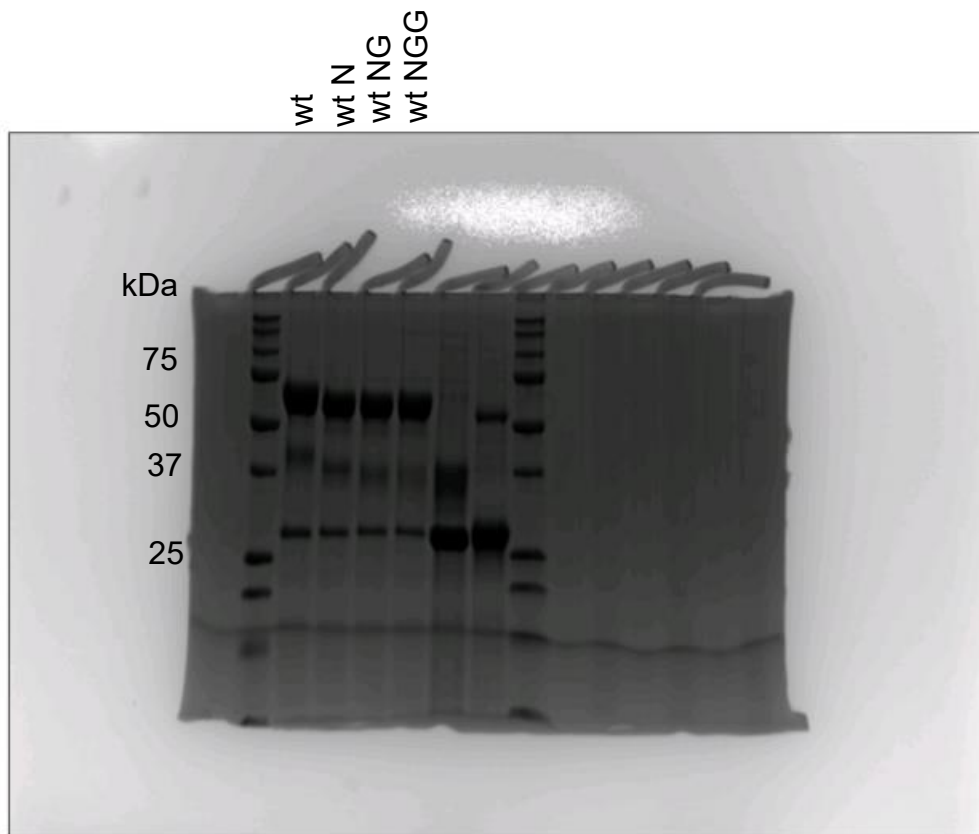

Supplement: Figure 2—figure supplement 1—source data 2. [file elife-100083-fig2-figsupp1-data2.zip › Figure S2 source data 2.pdf]
